# Two new species of Hymenochaetaceae from tropical Asia and America

**DOI:** 10.3389/fcimb.2022.1100044

**Published:** 2023-01-19

**Authors:** Meng Zhou, Xiao-Hong Ji, Hong-Gao Liu, Kurt Miller, Yuan Yuan, Josef Vlasák

**Affiliations:** ^1^ Institute of Microbiology, School of Ecology and Nature Conservation, Beijing Forestry University, Beijing, China; ^2^ College of Pharmacy and Life Sciences, Jiujiang University, Jiujiang, China; ^3^ Faculty of Agronomy and Life Sciences, Zhaotong University, Zhaotong, China; ^4^ Urb. Bellas Lomas, Mayaguez, Puerto Rico; ^5^ Biology Centre of the Academy of Sciences of the Czech Republic, České Budějovice, Czechia

**Keywords:** white rot, Hymenochaetaceae, polypore, taxonomy, wood-decaying fungi

## Abstract

Two new species in Hymenochaetaceae, *Fulvifomes acaciae* and *Pyrrhoderma nigra*, are illustrated and described from tropical Asia and America based on morphology and phylogenetic analyses. *F. acaciae* is characterized by perennial, pileate, and woody hard basidiomata when fresh; ash gray to dark gray, encrusted, concentrically sulcate, and irregularly cracked pileal surface; circular pores of 7–8 per mm with entire dissepiments; a dimitic hyphal system in trama and context; absence of setal element and presence of cystidioles; and broadly ellipsoid, yellowish brown, thick-walled, and smooth basidiospores measuring 5–6 μm × 4–5 μm. *P. nigra* is characterized by perennial and resupinate basidiomata with dark gray to almost black pore surface when fresh; small and circular pores of 7–9 per mm, a monomitic hyphal system with generative hyphae simple septate, hyphoid setae dominant in subiculum but not in tube trama, and absence of cystidia; and ellipsoid, hyaline, thin-walled basidiospores measuring 4–5 μm × 3–3.6 μm. The differences between the new species and morphologically similar and phylogenetically related species are discussed. Keys to *Fulvifomes* and *Pyrrhoderma* have also been provided.

## 1 Introduction

*Fulvifomes* is a monophyletic genus in Hymenochaetaceae (Wagner and Fischer, 2002; Wu et al., 2022). However, it has been treated as a synonym of *Phellinus* Quél. for several decades (Ryvarden and Johansen, 1980; Gilbertson and Ryvarden, 1986–1987a; Larsen and Cobb-Poulle, 1990; Núñez and Ryvarden, 2000). The genus is characterized by basidiomata annual to perennial, effused-reflexed; pileate or substipitate, corky to woody hard; hyphal system monomitic or dimitic; generative hyphae simple septate; setal elements absent; basidiospores subglobose to ellipsoid, yellowish to brown, fairly thick- to thick-walled, smooth; mostly on angiosperms and cause a white rot (Wu et al., 2022a). Recently, molecular analyses have detected new taxa in the genus (2015c; 2022b; Zhou, 2014; Ji et al., 2017; Salvador-Montoya et al., 2018; Wu et al., 2022a). So far, 49 species have been recorded in the genus (Wu et al., 2022a).

*Pyrrhoderma* Imazeki is another monophyletic genus in Hymenochaetaceae (Wu et al., 2022a) and was erected with *Pyrrhoderma sendaiense* as the generic type by Imazeki (1966). The genus was emended by Zhou et al. (2018). It is characterized by basidiomata annual to perennial, effused-reflexed, pileate to laterally stipitate, corky to woody hard when dry; pileal surface with a cuticle or crust; hyphal system monomitic; generative hyphae simple septate; hyphoid and hymenial setae present or absent; basidiospores ellipsoid to subglobose, hyaline, thin-walled; on angiosperm wood and cause a white rot. Previously, seven species were accepted in *Pyrrhoderma* (Wu et al., 2022a).

The pioneer phylogeny of Hymenochaetaceae was made by Wagner and Fischer (2001) based on limited samples, and more phylogenetic analyses were contributed based on more samples (2015b; 2016a; 2016b; Dai, 2010; Zhou, 2015a; Rajchenberg et al., 2015; Wu et al., 2016, 2022; Miettinen et al., 2019). Phylogenies of *Fulvifomes* were recently analyzed, and many new species were described (Zhou, 2015c; Ji et al., 2017; Tchoumi et al., 2020; Hattori, et al., 2022). *Pyrrhoderma* is a small genus in Hymenochaetaceae, and Zhou et al. (2018) published a comprehensive phylogeny on the genus.

During an investigation on tropical Asian and American hymenochaetaceous fungi, samples with morphological characteristics fit definitions of *Fulvifomes* and *Pyrrhoderma*. Phylogenetically, these have formed two distinct lineages within *Fulvifomes* and *Pyrrhoderma*, respectively. Therefore, in the present paper, we described two new species in Hymenochaetaceae.

## 2 Materials and methods

### 2.1 Morphological studies

Our studied specimens have been deposited in the herbarium of the Institute of Microbiology, Beijing Forestry University (BJFC), the private herbarium of Josef Vlasák (JV), and the National Museum Prague of Czech Republic (PRM). The sections were prepared in 5% potassium hydroxide (KOH), Melzer’s reagent (IKI), and cotton blue (CB). The following abbreviations are used: KOH, 5% potassium hydroxide; IKI, Melzer’s reagent; IKI–, neither amyloid nor dextrinoid; CB, cotton blue; CB–, acyanophilous; CB+, cyanophilous after 12 h stained with cotton blue; L, mean spore length (arithmetic average of spores); W, mean spore width (arithmetic average of spores); Q, variation in the ratios of L/W between specimens studied; and n, number of basidiospores measured from a given number of specimens. The microscopic procedure follows Dai (2010), and the special color terms follow Petersen (1996) and Anonymous (1969). Sections were studied at magnifications up to ×1,000 using a Nikon Eclipse 80i microscope with phase contrast illumination. Drawings were made with the aid of a drawing tube. Microscopic features, measurements, and illustrations were made from the slide preparations stained with CB. Basidiospores were measured from sections cut from the tubes.

### 2.2 DNA extraction, amplification, and sequencing

The extraction of total genomic DNA from frozen specimens followed Góes-Neto et al. (2005) using the protocol of Cetyltrimethyl Ammonium Bromide (CTAB) 2%. The CTAB rapid plant genome extraction kit-DN14 (Aidlab Biotechnologies Co., Ltd., Beijing) was used to obtain PCR products from dried specimens, following the manufacturer’s instructions with some modifications (2016; Chen et al., 2015). The internal transcribed spacer (ITS) region was amplified with the primer pairs ITS5 and ITS4 (White et al., 1990). For the large subunit nuclear ribosomal RNA gene (nLSU), the primer pairs LR0R and LR7 (Vilgalys and Hester, 1990) and LR0R and LR5 (White et al., 1990) were used for PCR amplification. The PCR procedure for ITS was as follows: initial denaturation at 95°C for 3 min, followed by 34 cycles of denaturation at 94°C for 40 s, annealing at 54°C for 45 s, and extension at 72°C for 1 min, followed by a final extension at 72°C for 10 min. The PCR procedure for 28S was as follows: initial denaturation at 94°C for 1 min followed by 35 cycles at 94°C for 30 s, 50°C for 1 min, 72°C for 1.5 min, and a final extension of 72°C for 10 min. The PCR products were purified and directly sequenced at Beijing Genomics Institute. The sequence quality was checked following Nilsson et al. (2012).

### 2.3 Phylogenetic analyses

**Figure 1:**
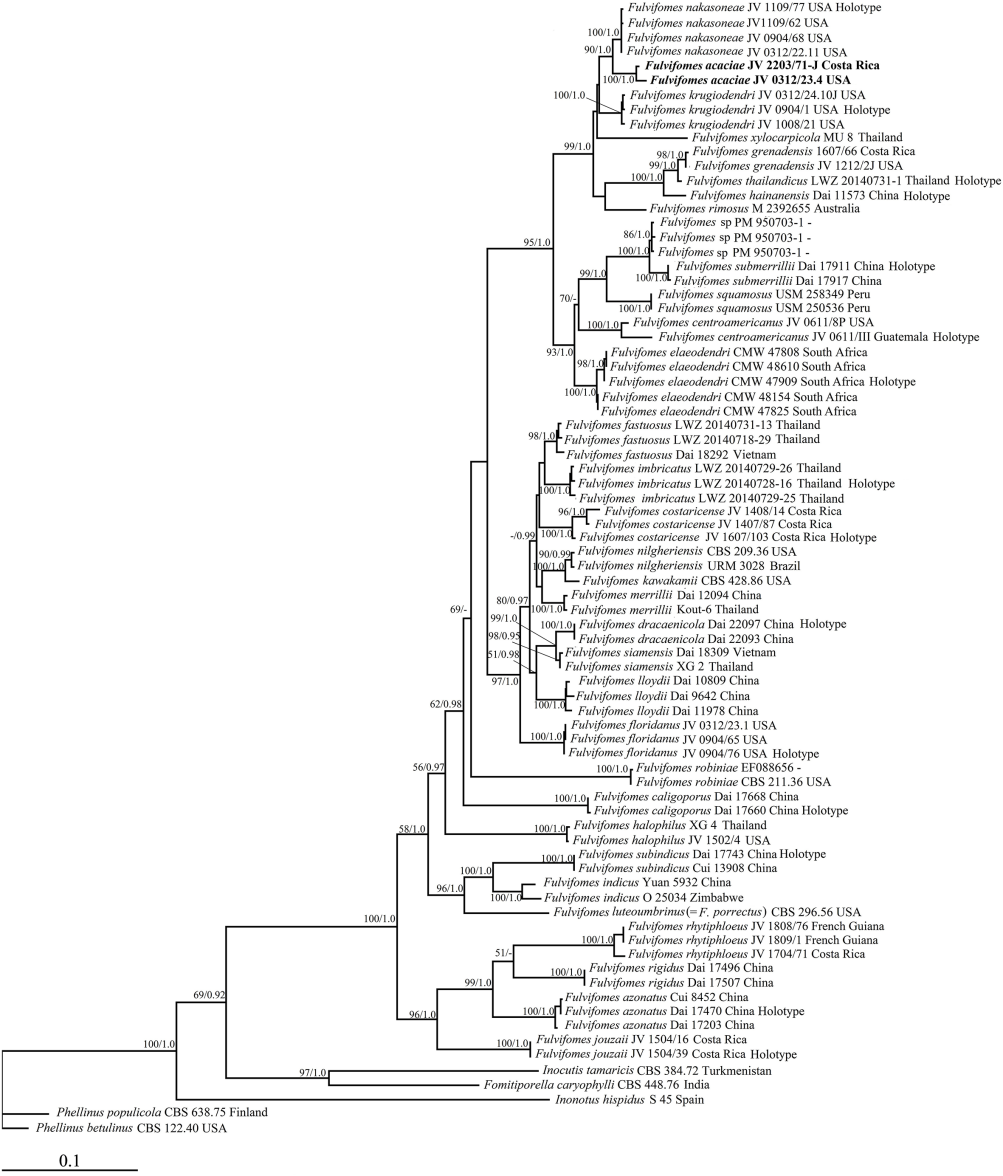
Maximum likelihood tree illustrating the phylogeny of *Fulvifomes* based on the combined dataset of ITS+28S sequences. *Phellinus betulinus* (MH856059; MH867554) and *P. populicola* (MH860960; MH872729) were used as outgroups. The maximum likelihood bootstrap values (≥50) and Bayesian posterior probability values (≥0.90) are indicated above the branches. The new species is in bold.

**Figure 2:**
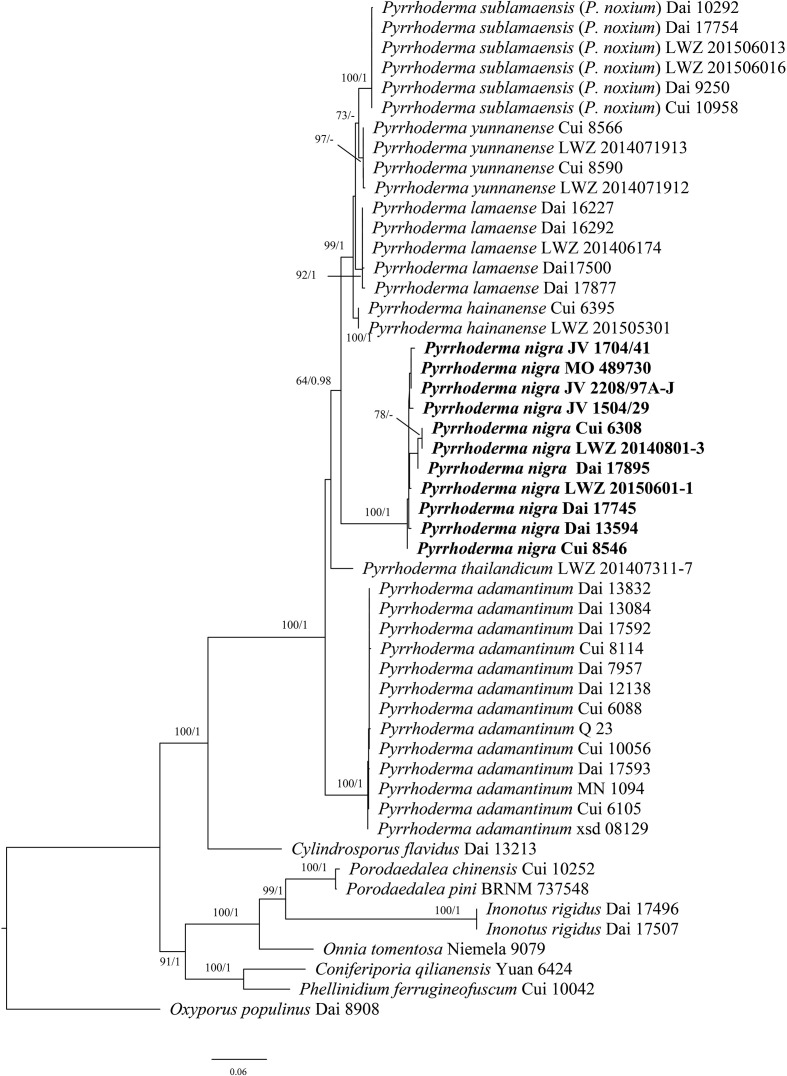
Maximum likelihood tree illustrating the phylogeny of *Pyrrhoderma* based on the combined dataset of ITS+28S sequences. *Oxyporus populinus* (KY131887; KT203323) was used as an outgroup. The maximum likelihood bootstrap values (≥50) and Bayesian posterior probability values (≥0.90) are indicated above the branches. The new species is in bold.

The phylogenetic trees were constructed using sequences obtained in this study and additional sequences downloaded from GenBank (**Tables 1**, **2**). Both ITS and 28S datasets were aligned within MAFFT version 7 (Katoh et al., 2019) and ClustalX (Thompson et al., 1997), followed by manual proofreading in BioEdit (Hall, 1999). Ambiguous regions were deleted, and gaps were manually adjusted to optimize alignment before phylogenetic analyses. *Phellinus betulinus* (Murrill) Parmasto and *P. populicola* Niemelä were used as outgroups in the phylogeny of *Fulvifomes* (Wu et al., 2022a; **Figure 1**). *Oxyporus populinus* (Schumach.) Donk was used as an outgroup in the phylogeny of *Pyrrhoderma* (Zhou et al., 2018; **Figure 2**). Each data matrix was edited in Mesquite version 3.70 (Maddison and Maddison, 2021).

**Table 1:** Taxa, voucher specimens, and GenBank accession numbers of sequences used in the phylogeny of *Fulvifomes*.

Species	Sample no.	Locality	GenBank accessions		Reference
			ITS	nLSU	
*Fomitiporella caryophylli*	CBS 448.76	India	AY558611	AY059021	Jeong et al., 2005
***Fulvifomes acaciae***	**JV 2203/71-J**	**Costa Rica**	**OP828594**	**OP828596**	**This study**
***F. acaciae***	**JV 0312/23.4**	**USA**	**OP828595**	**OP828597**	**This study**
*F. azonatus*	Cui 8452	China	MH390417	MH390396	Wu et al., 2022a
*F. azonatus*	Dai 17470	China	MH390418	MH390395	Wu et al., 2022a
*F. azonatus*	Dai 17203	China	MH390419	MH390397	Wu et al., 2022a
*F. caligoporus*	Dai 17668	China	MH390420	MH390390	Wu et al., 2022a
*F. caligoporus*	Dai 17660	China	MH390421	MH390391	Wu et al., 2022a
*F. centroamericanus*	JV 0611/III	Guatemala	KX960763	KX960764	Ji et al., 2017
*F. centroamericanus*	JV 0611/8P	USA	KX960757	N/A	Ji et al., 2017
*F. costaricense*	JV 1407/87	Costa Rica	MH390412	MH390387	Wu et al., 2022a
*F. costaricense*	JV 1408/14	Costa Rica	MH390413	MH390385	Wu et al., 2022a
*F. costaricense*	JV 1607/103	Costa Rica	MH390414	MH390386	Wu et al., 2022a
*F. dracaenicola*	Dai 22097	China	MW559800	MW559805	Du et al., 2021
*F. dracaenicola*	Dai 22093	China	MW559799	MW559804	Du et al., 2021
*F. elaeodendri*	CMW 47808	South Africa	MH599093	MH599131	Wu et al., 2022a
*F. elaeodendri*	CMW 47825	South Africa	MH599094	MH599134	Wu et al., 2022a
*F. elaeodendri*	CMW 47909	South Africa	MH599096	MH599132	Wu et al., 2022a
*F. elaeodendri*	CMW 48154	South Africa	MH599097	MH599135	Wu et al., 2022a
*F. elaeodendri*	CMW 48610	South Africa	MH599095	MH599133	Wu et al., 2022a
*F. fastuosus*	LWZ 20140731-13	Thailand	KR905674	KR905668	Zhou, 2015c
*F. fastuosus*	LWZ 20140718-29	Thailand	KR905673	N/A	Zhou, 2015c
*F. fastuosus*	Dai 18292	Vietnam	MH390411	MH390381	Wu et al., 2022a
*F. floridanus*	JV 0904/65	USA	MH390422	N/A	Wu et al., 2022a
*F. floridanus*	JV 0312/23.1	USA	MH390423	N/A	Wu et al., 2022a
*F. floridanus*	JV 0904/76	USA	MH390424	MH390388	Wu et al., 2022a
*F. grenadensis*	JV 1212/2J	USA	KX960756	N/A	Ji et al., 2017
*F. grenadensis*	1607/66	Costa Rica	KX960758	N/A	Ji et al., 2017
*F. hainanensis*	Dai 11573	China	KC879263	JX866779	Zhou, 2014
*F. halophilus*	XG 4	Thailand	JX104705	JX104752	KC879259
*F. halophilus*	JV 1502/4	USA	MH390427	MH390392	Wu et al., 2022a
*F. imbricatus*	LWZ 20140728-16	Thailand	KR905677	KR905670	Zhou, 2015c
*F. imbricatus*	LWZ 20140729-25	Thailand	KR905678	N/A	Zhou, 2015c
*F. imbricatus*	LWZ 20140729-26	Thailand	KR905679	KR905671	Zhou, 2015c
*F. indicus*	Yuan 5932	China	KC879261	JX866777	Zhou, 2014
*F. indicus*	O 25034	Zimbabwe	KC879262	KC879259	Wu et al., 2022a
*F. jouzaii*	JV 1504/16	Costa Rica	MH390425	MH390400	Wu et al., 2022a
*F. jouzaii*	JV 1504/39	Costa Rica	MH390426	N/A	Wu et al., 2022a
*F. kawakamii*	CBS 428.86	USA	N/A	AY059028	Wagner and Fischer, 2002
*F. krugiodendri*	JV 0904/1	USA	KX960762	KX960765	Ji et al., 2017)
*F. krugiodendri*	JV 0312/24.10J	USA	KX960760	KX960766	Ji et al., 2017)
*F. krugiodendri*	JV1008/21	USA	KX960761	KX960767	Ji et al., 2017)
*F. lloydii*	Dai 10809	China	MH390428	MH390378	Wu et al., 2022a
*F. lloydii*	Dai 9642	China	MH390429	MH390379	Wu et al., 2022a
*F. lloydii*	Dai 11978	China	MH390430	MH390380	Wu et al., 2022a
*F. luteoumbrinus*	CBS 296.56	USA	AY558603	AY059051	Wagner and Fischer, 2002
*F. merrillii*	Dai 12094	China	MH390415	MH390382	Wu et al., 2022a
*F. merrillii*	Kout-6	Thailand	MH390416	MH390383	Wu et al., 2022a
*F. nakasoneae*	JV 1109/62	USA	MH390407	MH390376	Wu et al., 2022a
*F. nakasoneae*	JV 0904/68	USA	MH390408	MH390373	Wu et al., 2022a
*F. nakasoneae*	JV 1109/77	USA	MH390409	MH390374	Wu et al., 2022a
*F. nakasoneae*	JV 0312/22.11	USA	MH390410	MH390375	Wu et al., 2022a
*F. nilgheriensis*	CBS 209.36	USA	AY558633	AY059023	Wagner and Fischer, 2002
*F. nilgheriensis*	URM 3028	Brazil	MH390431	MH390384	Wu et al., 2022a
*F. rhytiphloeus*	JV 1704/71	Costa Rica	MZ506738	MZ505207	Wu et al., 2022a
*F. rhytiphloeus*	JV 1808/76	French Guiana	MZ506739	MZ505208	Wu et al., 2022a
*F. rhytiphloeus*	JV 1809/10	French Guiana	MZ506740	MZ505209	Wu et al., 2022a
*F. rigidus*	Dai 17496	China	MH390432	MH390398	Wu et al., 2022a
*F. rigidus*	Dai 17507	China	MH390433	MH390399	Wu et al., 2022a
*F. rimosus*	M 2392655	Australia	MH628255	MH628017	Wu et al., 2022a
*F. robiniae*	CBS 211.36	USA	AY558646	AF411825	Wagner and Ryvarden, 2002
*F. robiniae*	Unknown	Unknown	EF088656	N/A	GenBank
*F. siamensis*	XG 2	Thailand	JX104709	JX104756	Zhou, 2014
*F. siamensis*	Dai 18309	Vietnam	MH390434	MH390389	Wu et al., 2022a
*F.* sp.	PM 950703-1	Unknown	EU035311	N/A	GenBank
*F.* sp.	PM 950703-1	Unknown	EU035312	N/A	GenBank
*F.* sp.	PM 950703-1	Unknown	EU035313	N/A	GenBank
*F. squamosus*	USM 250536	Peru	MF479268	MF479265	Salvador-Montoya et al., 2018
*F. squamosus*	USM 258349	Peru	MF479269	MF479264	Salvador-Montoya et al., 2018
*F. subindicus*	Dai 17743	China	MH390435	MH390393	Wu et al., 2022a
*F. subindicus*	Cui 13908	China	MH390436	MH390394	Wu et al., 2022a
*F. submerrillii*	Dai 17911	China	MH390405	MH390371	Wu et al., 2022a
*F. submerrillii*	Dai 17917	China	MH390406	MH390372	Wu et al., 2022a
*F. thailandicus*	LWZ 20140731-1	Thailand	KR905672	KR905665	Zhou, 2015c
*F. xylocarpicola*	MU 8	Thailand	JX104676	JX104723	Zhou, 2014
*Inocutis tamaricis*	CBS 384.72	Turkmenistan	AY558604	MH872221	Vu et al., 2018
*Inonotus hispidus*	S 45	Spain	EU282482	EU282484	GenBank
*Phellinus betulinus* (Outgroup)	CBS 122.40	USA	MH856059	MH867554	Wu et al., 2022a
*P. populicola* (Outgroup)	CBS 638.75	Finland	MH860960	MH872729	Wu et al., 2022a

New taxon is in bold. N/A, Not applicable.

**Table 2:** Taxa, voucher specimens, and GenBank accession numbers of sequences used in the phylogeny of *Pyrrhoderma*.

Species	Sample no.	Locality	GenBank accession no.		Reference
			ITS	nLSU	
*Coniferiporia qilianensis*	Yuan 6424	China	NR158318	NG060411	Zhou et al., 2016a
*Cylindrosporus flavidus*	Dai 13213	China	KP875564	KP875561	Zhou, 2015a
*Inonotus rigidus*	Dai 17496	China	MH390432	MH390398	GenBank
*I. rigidus*	Dai 17507	China	MH390433	MH390399	GenBank
*Onnia tomentosa*	Niemela 9079	Finland	MF319075	MF318931	GenBank
*Phellinidium ferrugineofuscum*	Cui 10042	China	KR350573	KR350559	Zhou et al., 2016a
*Porodaedalea chinensis*	Cui 10252	China	KX673606	MH152358	Dai et al., 2017
*P. pini*	BRNM 737548 (CFMR)	Turkey	JQ772470	N/A	Tomsovsky and Kout, 2013
*P. adamantinum*	Cui 6088	Jiangxi, China	MF860783	N/A	Zhou et al., 2018
*P. adamantinum*	Cui 6105	Jiangxi, China	MF860784	MF860733	Zhou et al., 2018
*P. adamantinum*	Cui 8114	Yunnan, China	MF860785	MF860734	Zhou et al., 2018
*P. adamantinum*	Cui 10056	Jilin, China	MF860786	N/A	Zhou et al., 2018
*P. adamantinum*	Dai 7957	Hainan, China	MF860787	N/A	Zhou et al., 2018
*P. adamantinum*	Dai 12138	Hunan, China	MF860788	N/A	Zhou et al., 2018
*P. adamantinum*	Dai 13084	Yunnan, China	MF860789	MF860735	Zhou et al., 2018
*P. adamantinum*	Dai 13832	Yunnan, China	MF860790	MF860736	Zhou et al., 2018
*P. adamantinum*	Dai 17592	Yunnan, China	MF860791	MF860737	Zhou et al., 2018
*P. adamantinum*	Dai 17593	Yunnan, China	MF860792	MF860738	Zhou et al., 2018
*P. adamantinum*	MN 1094	Japan	N/A	AY059031	Wagner and Fischer, 2002
*P. adamantinum*	Q 23	China	KC414229	N/A	GenBank
*P. adamantinum*	xsd 08129	China	FJ481040	N/A	GenBank
*P. hainanense*	Cui 6395	Hainan, China	MF860793	N/A	Zhou et al., 2018
*P. hainanense*	LWZ 20150530-1	Hainan, China	MF860794	MF860739	Zhou et al., 2018
*P. lamaoense*	Dai 16227	Hainan, China	MF860802	MF860743	Zhou et al., 2018
*P. lamaoense*	Dai 16292	Hainan, China	MF860803	MF860744	Zhou et al., 2018
*P. lamaoense*	Dai 17500	Yunnan, China	MF860804	MF860748	Zhou et al., 2018
*P. lamaoense*	Dai 17877	Singapore	MF860805	MF860749	Zhou et al., 2018
*P. lamaoense*	LWZ 20140617-4	Guangxi, China	MF860806	MF860746	Zhou et al., 2018
***P. nigra***	**Cui 6308**	**Hainan, China**	**N/A**	**MF860757**	Zhou et al., 2018
***P. nigra***	**Cui 8546**	**Yunnan, China**	**MF860816**	**MF860758**	Zhou et al., 2018
***P. nigra***	**Dai 13594**	**Yunnan, China**	**N/A**	**MF860759**	Zhou et al., 2018
***P. nigra***	**Dai 17745**	**Hainan, China**	**N/A**	**MF860760**	Zhou et al., 2018
***P. nigra***	**Dai 17895**	**Singapore**	**N/A**	**MF860761**	Zhou et al., 2018
***P. nigra***	**JV1504/29**	**Costa Rica**	**MF860817**	**MF860762**	Zhou et al., 2018
***P. nigra***	**JV1704/41**	**Costa Rica**	**MF860818**	**MF860763**	Zhou et al., 2018
***P. nigra***	**JV 2208/97A-J**	**French Guiana**	**OP824782**	**N/A**	**This study**
***P. nigra***	**LWZ 20140801-3**	**Thailand**	**MF860819**	**MF860764**	Zhou et al., 2018
***P. nigra***	**LWZ 20150601-1**	**Hainan, China**	**MF860820**	**MF860765**	Zhou et al., 2018
***P. nigra***	**MO 489730**	**Puerto Rico**	**OP605521**	**N/A**	**This study**
*P. sublamaensis (P. noxium)*	Cui 10958	Hainan, China	MF860807	N/A	Zhou et al., 2018
*P. sublamaensis (P. noxium)*	Dai 9250	Hainan, China	MF860808	N/A	Zhou et al., 2018
*P. sublamaensis (P. noxium)*	Dai 10292	Hainan, China	KX058573	HQ328532	Dai, 2010
*P. sublamaensis (P. noxium)*	Dai 17754	Hainan, China	MF860809	MF860752	Zhou et al., 2018
*P. sublamaensis (P. noxium)*	LWZ 20150601-3	Hainan, China	MF860810	MF860750	Zhou et al., 2018
*P. sublamaensis (P. noxium)*	LWZ 20150601-6	Hainan, China	MF860811	MF860751	Zhou et al., 2018
*P. thailandicum*	LWZ 20140731-17	Thailand	MF860812	MF860753	Zhou et al., 2018
*P. yunnanense*	Cui 8566	Yunnan, China	MF860813	N/A	Zhou et al., 2018
*P. yunnanense*	Cui 8590	Yunnan, China	N/A	MF860754	Zhou et al., 2018
*P. yunnanense*	LWZ 20140719-12	Yunnan, China	MF860814	MF860755	Zhou et al., 2018
*P. yunnanense*	LWZ 20140719-13	Yunnan, China	MF860815	MF860756	Zhou et al., 2018
*Oxyporus populinus* (Outgroup)	Dai 8908	China	KY131887	KT203323	Wu et al., 2017

New taxon is in bold. N/A, Not applicable.

Phylogenetic analyses were conducted using maximum likelihood (ML) and Bayesian Inference (BI) based on ITS+28S aligned datasets using RAxML version 8.2.12 (Stamatakis, 2014) and MrBayes version 3.2.6 (Ronquist et al., 2012). Sequence alignments were deposited at TreeBase (http://purl.org/phylo/treebase; submission ID 29762 and 29862).

GTR+I+G was estimated as the best-fit evolutionary model for the resulting alignments from these two datasets with jModelTest (Guindon and Gascuel, 2003; Posada, 2008). RAxML version 8.2.12 (Stamatakis, 2014) was applied in the ML analysis. All parameters in the ML analysis were kept at default settings.

The BI was calculated with MrBayes version 3.2.6 (Ronquist et al., 2012) in two independent runs, each of which had four chains for 1.5 million generations that were initiated using random trees. Trees were sampled every 100 generations. The first 25% of the sampled trees were discarded as burn-in, whereas other trees were used to construct a 50% majority consensus tree and for calculating Bayesian posterior probabilities (BPPs).

The two methods constructed nearly congruent topologies for each alignment. Therefore, only the topology generated from the ML analysis is presented along with the bootstrap support for ML (BS) values and BPPs, simultaneously at the nodes. Phylogenetic trees were visualized using FigTree version 1.4.4 (Rambaut, 2018). Branches that received bootstrap support for ML (BS) and BPPs (≥75% for BS and 0.95 for BPPs) were considered as significantly supported.

## 3 Results

### 3.1 Phylogeny

Permission in the phylogenetic analysis of *Fulvifomes* (**Figure 1**), 79 fungal collections representing 37 taxa of *Fulvifomes* were included in the phylogenetic analyses and two samples of genus *Phellinus* were used as outgroups. The final alignment comprised a total of 1,922 base pairs (bp), including 1,032 bp of ITS and 890 bp of 28S. The best model for the combined ITS+28S dataset was estimated and applied in the Bayesian analysis: GTR+I+G, lset nst = 6, rates = invgamma; prset statefreqpr = dirichlet (1,1,1,1). Bayesian analysis resulted in an average standard deviation of split frequencies as 0.009512. As both ML and BI trees resulted in similar topologies, only the topology from the ML analysis is presented along with statistical values from the ML (≥50%) and BPP (≥0.9) algorithms (**Figure 1**).

In the phylogenetic analysis of *Pyrrhoderma* (**Figure 2**), the ITS+28S sequences from 51 fungal collections representing 15 species were used. The final alignment comprised a total of 1,686 bp, including 814 bp of ITS and 872 bp of 28S. The best model for the combined ITS+28S dataset was estimated and applied in the Bayesian analysis: GTR+I+G, lset nst = 6, rates = invgamma; prset statefreqpr = dirichlet (1,1,1,1). Bayesian analysis resulted in an average standard deviation of split frequencies = 0.006184. Both ML and BI trees resulted in similar topologies; thus, only the topology from the ML analysis is presented along with statistical values from the ML (≥50%) and BPP (≥0.9) algorithms (**Figure 2**).

### 3.2 Taxonomy

**Figure 3:**
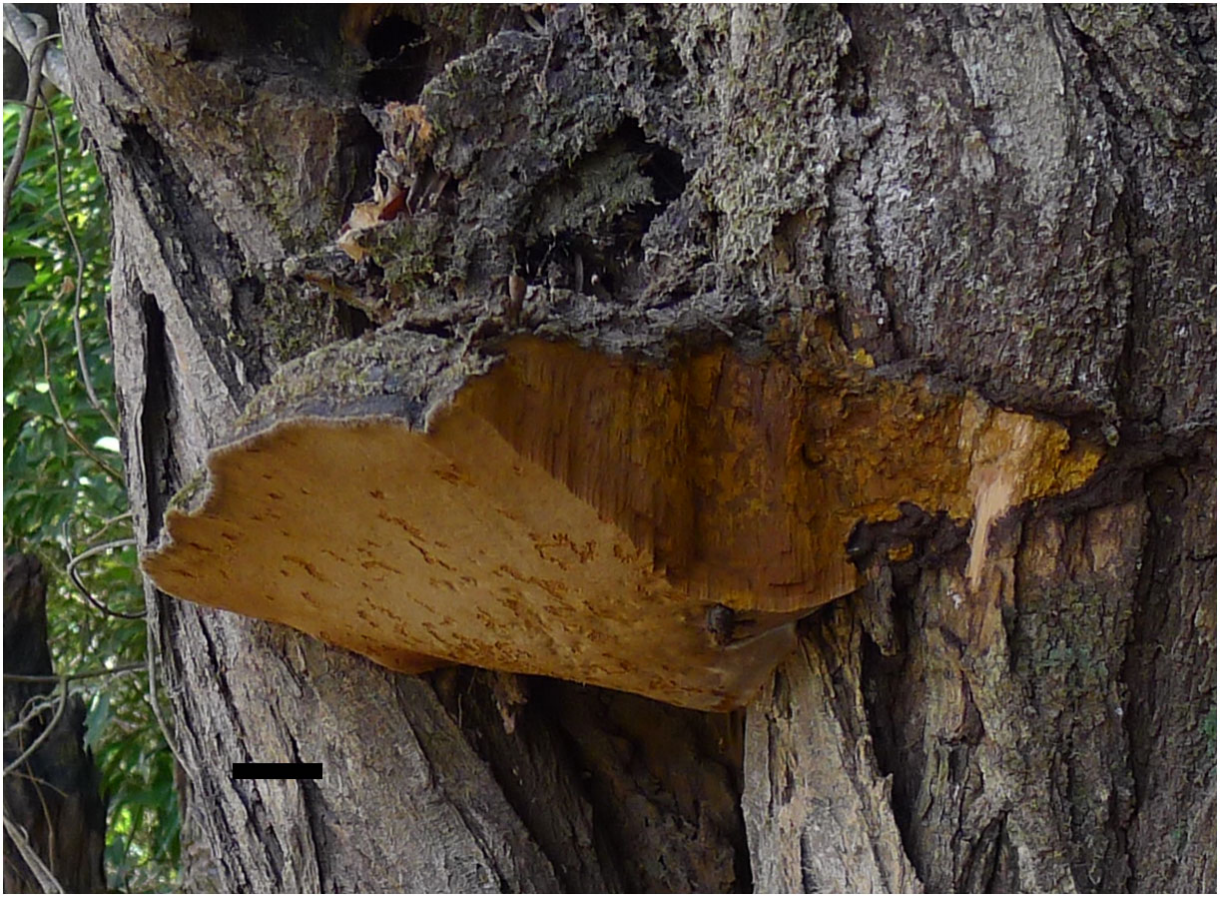
A basidiomata of *Fulvifomes acaciae* (holotype, JV 2203/71-J). Scale bar: 1 cm.

**Figure 4:**
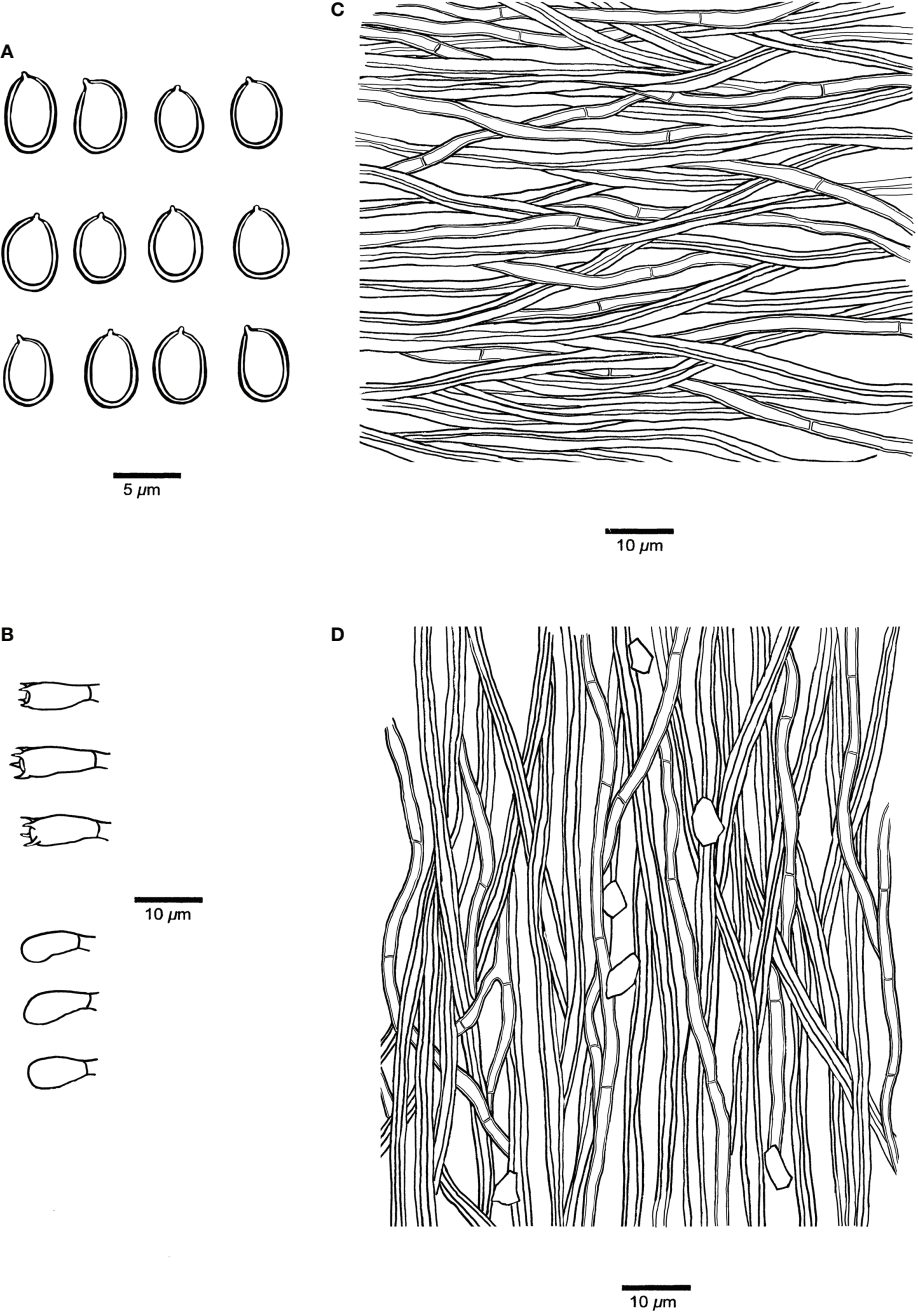
Microscopic structures of *Fulvifomes acaciae* (JV 0312/23.4-J). **(A)** Basidiospores. **(B)** Basidia and basidioles. **(C)** Hyphae from context. **(D)** Hyphae from tube trama.

***Fulvifomes acaciae*** Meng Zhou, Yuan & Vlasák, sp. **nov.** **Figures 3**, **4**.

MycoBank: MB xxxxxx

**Type**. Costa Rica, Mt. Rincon, Guachipelin, on living tree of *Acacia*, March 2022, JV 2203/71-J (isotype, BJFC).

**Etymology**. *Acaciae* (Lat.): referring to the species growing on *Acacia*.

**Fruiting body.** Basidiomata perennial, pileate, solitary, without distinctive odor or taste and woody hard when fresh, light in weight when dry. Pilei ungulate, projecting up to 20 cm and 15 cm wide and 7 cm thick at base. Pileal surface ash gray to dark gray when dry, encrusted, rough, concentrically sulcate, irregularly cracked; pileal margin dark gray, obtuse. Pore surface umber, glancing; sterile margin distinct, fulvous, up to 3 mm wide; pores circular, 7–8 per mm; dissepiments thick, entire. Context fulvous, woody hard, zonate, up to 5 mm thick. Tubes concolorous with context, woody hard, up to 6.5 cm long, tube layers indistinctly stratified.

**Hyphal structure.** Hyphal system dimitic in trama and context; generative hyphae simple septate; tissue becoming blackish brown in KOH.

**Context.** Generative hyphae hyaline to pale yellow, thin- to thick-walled, rarely branched, frequently simple septate, 2–3 μm in diameter; skeletal hyphae dominant, yellowish to golden yellow, thick-walled with a narrow to wide lumen, unbranched, aseptate, more or less straight, regularly arranged, 3–4 μm in diameter.

**Tubes.** Generative hyphae hyaline to pale yellow, thin- to slightly thick-walled, rarely branched, frequently simple septate, 2–3.5 μm in diameter; skeletal hyphae frequent, yellowish to golden yellow, thick-walled with a narrow to wide lumen, unbranched, aseptate, more or less straight, subparallel along tubes, 3–4.5 μm in diameter. Setae or setal hyphae absent; cystidioles absent; basidia barrel-shaped, with four sterigmata and a simple basal septum, 10–12 μm × 5–6 μm; basidioles in shape similar to basidia, slightly smaller than basidia. Big rhomboid crystals present in hymenia and trama.

**Spores**. Basidiospores broadly ellipsoid, yellowish brown, thick-walled, smooth, some collapsed, IKI–, CB–, (4.9–)5–6(–6.1) μm × (3.9–)4–5(–5.1) μm, L = 5.26 μm, W = 4.35 μm, Q = 1.19–1.23 (n = 60/2).

*Additional specimen (paratype) examined.* USA, Florida, Florida Keys, Key Largo, John Pennekamp Coral Reef State Park, December 2003, Josef Vlasák leg., on fallen trunk of *Acacia*, JV 0312/23.4-J (BJFC032898).

**Figure 5:**
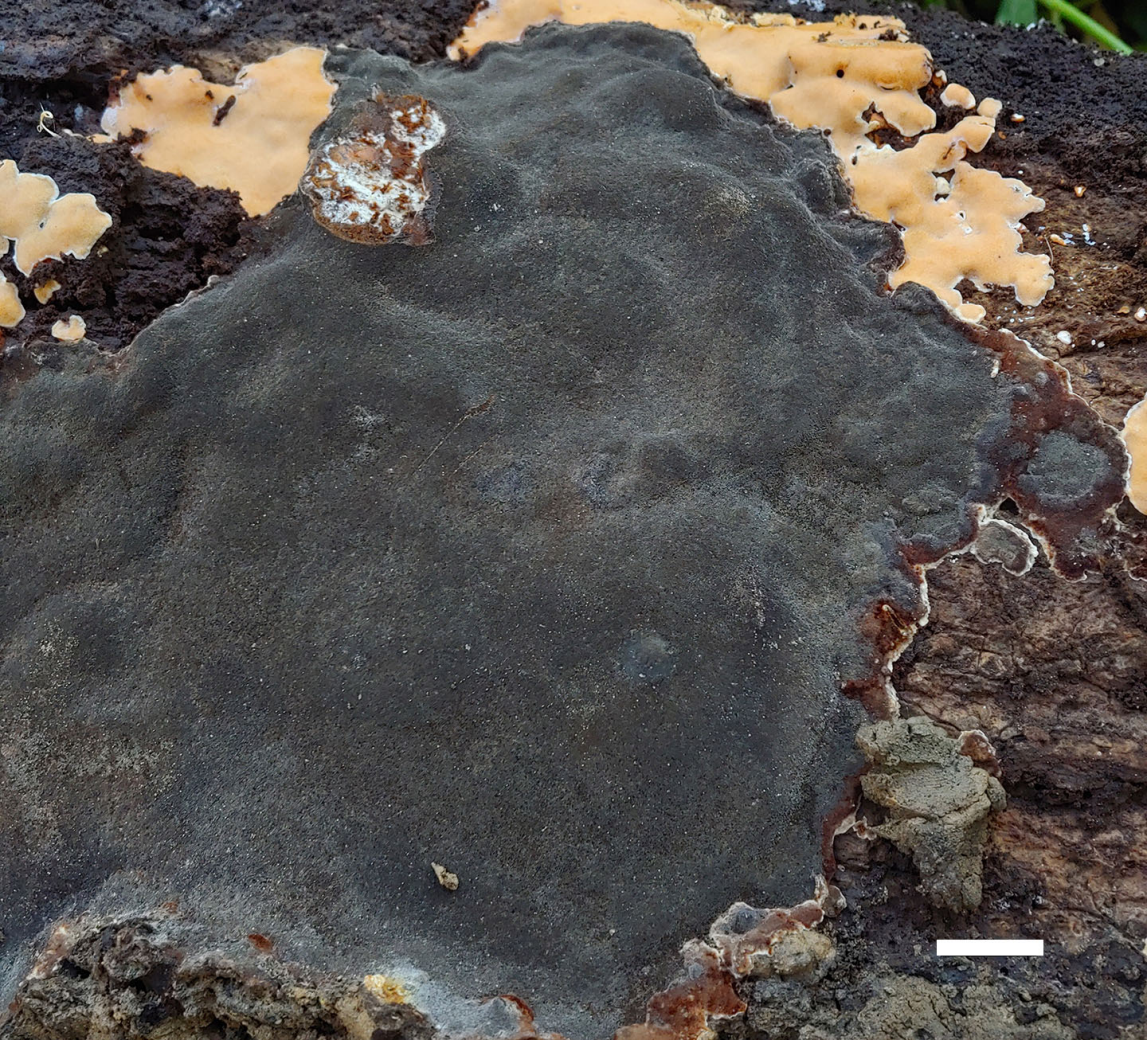
A basidiomata of *Pyrrhoderma nigra* (MO 489730). Scale bar: 1 cm.

**Figure 6:**
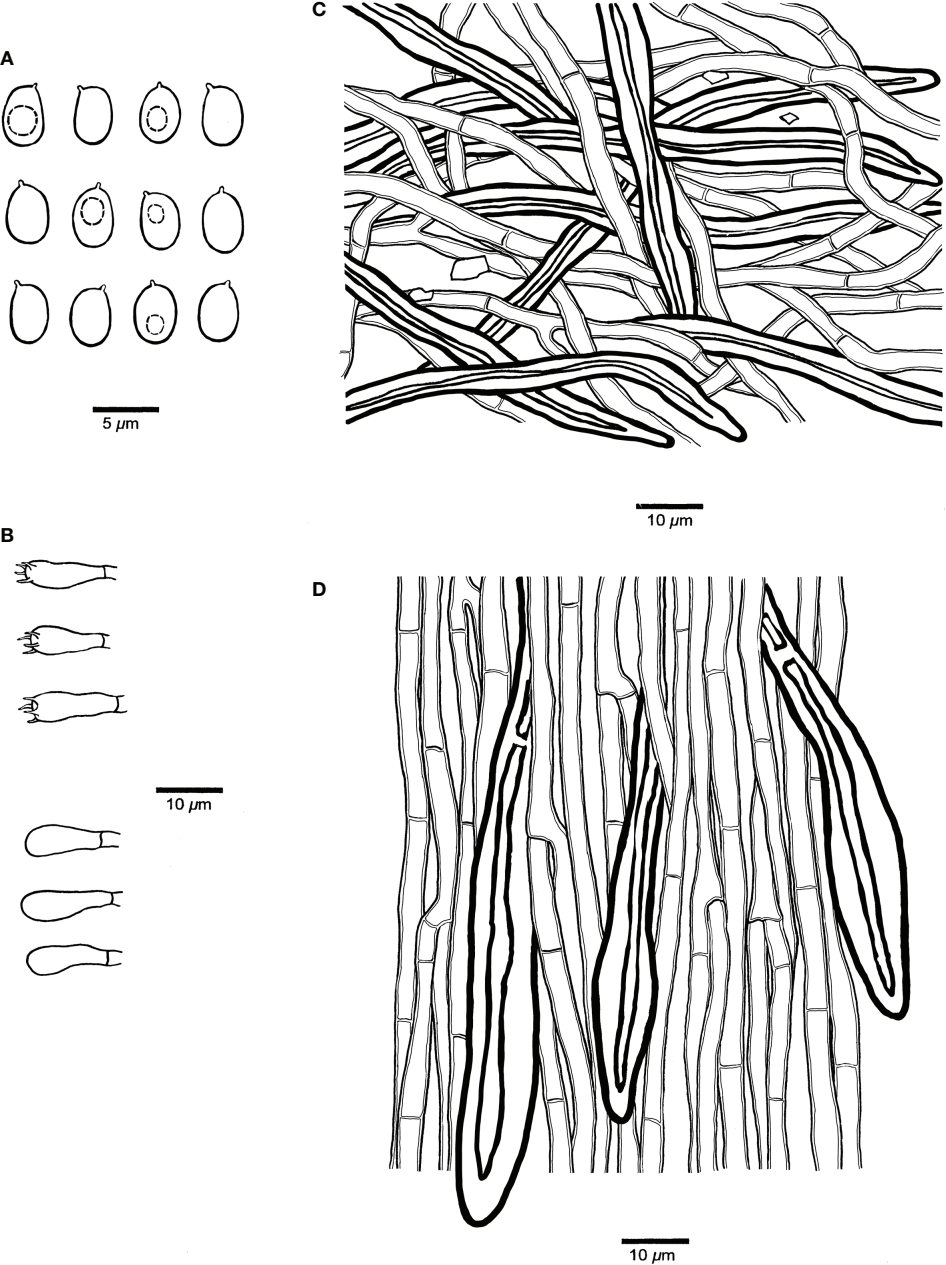
Microscopic structures of *Pyrrhoderma nigra* (holotype, Cui 8546). **(A)** Basidiospores. **(B)** Basidia and basidioles. **(C)** Hyphae from subiculum. **(D)** Hyphae from tube trama.

***Pyrrhoderma nigra*** Meng Zhou, Yuan Yuan & Vlasák, sp. nov. **Figures 5**, **6**

MycoBank: MB xxxxxx.

**Type.** China, Yunnan Province, Mengla County, Wangtianshu Forest Park, 2 November 2009, Bao-Kai Cui leg., on fallen angiosperm trunk, Cui 8546 (BJFC 007035).

**Etymology.** *Nigra* (Lat.): referring to having black pore surface when fresh.

**Fruiting body.** Basidiomata perennial, resupinate, firmly attached to the substrate, separable, up to 30 cm long, 16 cm wide and 8 mm thick at center, without odor or taste when fresh, woody hard and brittle when dry. Pore surface dark gray to almost black when fresh, becoming grayish brown when dry, glancing; sterile margin very narrow to almost absent, dark brown; pores mostly circular, 7–9 per mm; dissepiments thick, entire. Subiculum chestnut, woody hard, up to 3 mm thick. Tubes deep olive, woody hard to brittle, up to 3 mm long.

**Hyphal structure.** Hyphal system monomitic; generative hyphae simple septate; tissue darkening but otherwise unchanged in KOH.

**Subiculum**. Subicular hyphae yellowish to golden yellow, thick-walled with a wide lumen, occasionally branched, frequently simple septate, interwoven, some encrusted with fine crystals, 4–5.5 µm diameter; hyphoid setae dark brown, distinctly thick-walled with a narrow lumen, straight, apex obtuse and not encrusted, up to a few hundreds of µm long, 5–8 µm diameter.

**Tubes**. Tramal hyphae pale yellowish to yellow, thin- to thick-walled with a wide lumen, gelatinized, frequently branched, frequently simple septate, parallel along the tubes, 3–4.5 µm diameter; hyphoid setae frequent, but not dominant, dark brown, distinctly thick-walled with a narrow lumen, straight, apex obtuse or pointed, and sometimes encrusted with fine hyaline crystals, frequently projecting out of hymenium, up to a few hundreds of micrometers long, 8–12 µm diameter; cystidia and cystidioles absent; basidia barrel-shaped, with four sterigmata and a simple septum at the base, 7–9 µm × 4–4.5 µm; basidioles more or less pyriform, slightly smaller than basidia.

**Spores**. Basidiospores ellipsoid, hyaline, thin-walled, some with a big guttule, IKI–, CB–, 4–5 μm × 3–3.6(–4) μm, L = 4.43 µm, W = 3.34 µm, Q = 1.33 (n = 30/1).

*Additional specimens (paratypes) examined*. China: Hainan Province, Ledong County, Jianfengling National Nature Reserve, on fallen angiosperm trunk, 1 June 2015, LWZ 20150601-1 (IFP 019170). Costa Rica, Golfito, Playa Cacao, 19.IV.2015, JV 1504/29 (JV), Playa Nicuesa, 18.IV.2017, JV 1704/41(JV). French Guiana, Roura, Camp Cayman, 27.VIII.2018, JV 1808/107 (BJFC032959), St. Laurent du Maroni, Gite Moutouchi, on fallen angiosperm trunk, 31.VIII.2022, JV 2208/97A-J. (JV, BJFC) Puerto Rico, Mayagüez, Miradero, Papaya House, on fallen mango trunk, 4.8.2022, Kurt Miller MO 489730 (JV, BJFC).

## 4 Discussion

Macromorphologically, *Fulvifomes krugiodendri* has perennial, solitary, ungulate basidiomata; its pileal surface is dark gray, encrusted, concentrically sulcate with narrow zones, cracked with age; its pores as 7–9 per mm with thick and entire dissepiments. Microscopically, it has a dimitic hyphal system in both context and tube trama. Morphologically, *F. krugiodendri* is similar to *F. acaciae*, and both species are also closely related in our phylogeny (**Figure 1**). However, *F. krugiodendri* differs from *F. acaciae* by its subglobose basidiospores measuring 4.3–5 μm × 4–4.5 μm, L = 4.6 μm, W = 4.21 μm, Q = 1.08–1.09, interwoven tramal hyphae, and the absence of rhomboid crystals, and it lives on *Krugiodendron* (Ji et al., 2017). In addition, the nucleotide difference of ITS sequences between the two species is 3%.

Morphologically, *Pyrrhoderma nigra* is very similar to the resupinate *Pyrrhoderma lamaoense* (Murrill) L.W. Zhou & Y.C. Dai and *P. sublamaensis* (Lloyd) Y.C. Dai & F. Wu, but the latter two species have effused-reflexed to pileate basidiomata, the presence of cystidia, especially thinner basidiospores (2–2.4 μm *vs*. 3–3.6 µm, Wu et al., 2022). *P. nigra* and *Pyrrhoderma thailandicum* L. W. Zhou & Y.C. Dai share similar basidiospores (4–5 µm × 3–3.6 µm *vs*. 4–4.5 µm × 3–3.5 µm), but the latter differs from the former by annual basidiomata, bigger pores (3–5 per mm *vs*. 7–9 per mm) and the absence of setal elements.

Most species of *Fulvifomes* and *Pyrrhoderma* have been recorded in the tropics (Wu et al., 2022). The two new species described in the present study were found from tropical Asia and America. Similar to other polypores, species of Hymenochaetaceae is very rich in the tropics (Dai et al., 2021). So far, 50 and 8 species of *Fulvifomes* and *Pyrrhoderma*, respectively, have been identified, and identification keys to the species in the two genera are given below.

**Key to species of *Fulvifomes***

1. Basidiocarps annual ..........................................................................2

1. Basidiocarps perennial .....................................................................5

2. Pores 4–5 per mm ....................*F. indicus* (Massee) L. W. Zhou

2. Pores 7–10 per mm ..........................................................................3

3. Basidiocarps resupinate; basidiospores ellipsoid...........................................*F. rigidus* (B.K. Cui & Y.C. Dai) X.H. Ji & Jia J. Chen

3. Basidiocarps pileate; basidiospores globose to subglobose......... 4

4. Pileal surface without a black cuticle; hyphal system dimitic........ *F. aureobrunneus* (J.E. Wright & Blumenf.) Y.C. Dai & F. Wu

4. Pileal surface with a black cuticle; hyphal system monomitic.............................*F. luteoumbrinus* (Romell) Y. C. Dai et al.

5. Chlamydospores present.................................................................. 6

5. Chlamydospores absent.....................................................................9

6. Basidiospores 5–8 µm long............................................................... 7

6. Basidiospores 4–5 µm long............................................................... 8

7. Pilei ungulate, tube layers separated by a thin context layer..... *F. scaber (*Berk.) Y.C. Dai & F. Wu

7. Pilei globose, tubes indistinctly stratified without context layer..........*F. kravtzevii (*Schwarzman) Y.C. Dai & F. Wu

8. Basidiocarps imbricate, pileal surface with a cuticle....................*F. kawakamii* (M.J. Larsen et al.) T. Wagner & M. Fisch.

8. Basidiocarps solitary, pileal surface without a cuticle.................*F. durissimus* (Lloyd) Bondartseva & S. Herrera

9. Basidiospores oblong-ellipsoid.......................................................10

9. Basidiospores ellipsoid, broadly ellipsoid, ovoid, subglobose or globose.......................................................................................................11

10. Pores 5–6 per mm; basidiospores 4.2–5.1 µm long....... *F. collinus* (Y.C. Dai & Niemelä) Y.C. Dai

10. Pores 7–8 per mm; basidiospores 3–3.6 µm long.......................*F. fushanianus* (T.T. Chang) Y.C. Dai & F. Wu

11. Tramal hyphae monomitic...........................................................12

11. Tramal hyphae dimitic..................................................................14

12. Basidiospores ellipsoid, <4 µm wide............................................*F. caligoporus* Y.C. Dai & X.H. Ji

12. Basidiospores ovoid or subgolobose, >4 µm wide....................13

13. Context without a granular core, pores 5–7 per mm.....*F. lloydii* (Cleland) Y.C. Dai & X.H. Ji

13. Context with a granular core, pores 3–4 per mm......................*F. resinaceus* (Kotl. & Pouzar) Y.C. Dai & F. Wu

14. Hyphae monomitic in context.....................................................15

14. Hyphae dimitic or subdimitic in context....................................25

15. Pileal surface uncracked................................................................16

15. Pileal surface cracked or rimose...................................................20

16. Hyphae at pileal surface with thin-walled and septate tips; on *Newtonia buchananii*; African species.................................*F. newtoniae* (Niemelä & Mrema) Y.C. Dai & F. Wu

16. Hyphae at pileal surface without thin-walled and septate tips; on an angiosperm other than *Newtonia*; Asian or American species............................17

17. Basidiospores >5.5 μm long...................................*F. mangrovicus* (Imazeki) T. Hatt.

17. Basidiospores <5.5 μm long..........................................................18

18. On *Dracaena*................................................*F. dracaenicola* Z.B. Liu & Y.C. Dai

18. On an angiosperm other than *Dracaena*....................................19

19. Pore surface not glancing; basidiospores CB+; Asian species.......*F. subindicus* Y.C. Dai & X.H. Ji

19. Pore surface glancing; basidiospores CB–; American species........*F. floridanus* Y.C. Dai & Vlasák

20. Pileal surface squamose with long scales.........*F. squamosus* Salvador-Montoya & Drechsler-Santos

20. Pileal surface glabrous or tomentose without long scales........21

21. Basidiospores globose, >5 µm wide...............................*F. cedrelae* (Murrill) Murrill

21. Basidiospores ovoid, broadly ellipsoid to subglobose, <5 µm wide..........................................................................................................22

22. Pileal surface with a black crust....................................................23

22. Pileal surface without crust...........................................................24

23. Pores 4–7 per mm; basidiospores 3–4 µm wide.................*F. grenadensis* (Murrill) Murrill

23. Pores 7–8 per mm; basidiospores 4–5 µm wide......................*F. siamensis* T. Hatt. et al.

24. Pore surface dull chocolate brown, pores 4–5 per mm................*F. rimosus (*Berk.) Fiasson & Niemelä

24. Pore surface yellowish to reddish brown, pores 7–8 per mm.....................*F. robiniae* (Murrill) Murrill

25. Pileal surface azonate.....................................................................26

25. Pileal surface concentrically zonate.............................................28

26. Basidiospores 4.5–6 μm wide........................................*F. crocatus* (Fr.) Y.C. Dai & F. Wu

26. Basidiospores 3–4 μm wide..........................................................27

27. Pore surface not glancing, pores 7–9 per mm...........*F. azonatus* Y.C. Dai & X.H. Ji

27. Pore surface glancing, pores 5–7 per mm................*F. swieteniae* Murrill

28. Pileal surface cracked.....................................................................29

28. Pileal surface uncracked................................................................37

29. Pores 7–11 per mm.........................................................................30

29. Pores 4–7 per mm...........................................................................32

30. Pilei triquetrous, pore surface dark brown, not glancing.........*F. minutiporus* (Bond. & Herrera) Y.C. Dai & F. Wu

30. Pilei ungulate, pore surface grayish brown to umber, glancing............31

31. Basidiospores subglobose, 4.3–5 μm × 4–4.5 μm......................*F. krugiodendri* Y.C. Dai et al.

31. Basidiospores subglobose broadly ellipsoid, 5–6 μm × 4–5 μm................................................................................................................................ *F. acaciae*

32. Growing on *Pseudocedrela* or *Elaeodendron*; African species..............................................................................................33

32. Growing on an angiosperm other than *Pseudocedrela* and *Elaeodendron*; Asian and American species............................................34

33. Context with a black line; on *Elaeodendron croceum...............F. elaeodendri* Tchotet et al.

33. Context without a black line; on *Pseudocedrela kotschyi.........F. yoroui* Olou & F. Langer

34. Basidiospores mostly 5–6 μm wide......................*F. coffeatoporus* (Kotl. & Pouzar) Y.C. Dai & F. Wu

34. Basidiospores mostly 3.5–5 μm wide..........................................35

35. Pore surface not glancing; American species.............................*F. nakasoneae* Y.C. Dai & Vlasák

35. Pore surface glancing; Asian species...........................................36

36. Pileal surface matted, not encrusted; cystidioles absent...........*F. xylocarpicola* T. Hatt. et al.

36. Pileal surface encrusted; cystidioles present........*F. thailandicus* L.W. Zhou

37. Pores 3–4 per mm.................................*F. hainanensis* L.W. Zhou

37. Pores 4–11 per mm.........................................................................38

38. Cystidioles present.........................................................................39

38. Cystidioles absent...........................................................................41

39. A thin black line present........................................between context and substrate *F. allardii* (Bres.) Bondartseva & S. Herrera

39. A thin black line absent.................................................................40

40. Pores 4–5 per mm; basidiospores ellipsoid to reniform..........*F. merrillii* (Murrill) Baltazar & Gibertoni

40. Pores 6–7 per mm; basidiospores ellipsoid...........*F. submerrillii* X.H. Ji & Jia J Chen

41. Growing on *Abies;* context very thin to almost lacking............*F. acontextus* (Ryvarden) Y.C. Dai & F. Wu

41. Growing angiosperm wood; distinct context present..............42

42. Basidiocarps usually effused-reflexed to pileate........................43

42. Basidiocarps distinctly pileate......................................................44

43. Pileal surface dark brown to black; growing exclusively on *Xylocarpus........................................................F. halophilus* T. Hatt. et al.

43. Pileal surface luteous brown; growing on an angiosperm other than *Xylocarpus..........................................F. mcgregorii* (Bres.) Y.C. Dai

44. Basidiospores 5–6 µm long...........................................*F. fastuosus* (Lév.) Bondartseva & S. Herrera

44. Basidiospores 4–5 µm long...........................................................45

45. Basidiospores globose..............................................*F. rhytiphloeus* (Mont.) Camp.-Sant. & Robledo

45. Basidiospores broadly ellipsoid to subglobose..........................46

46. Pilei ungulate; basidiospores <3.7 μm wide...................*F. jouzaii* Y.C. Dai & F. Wu

46. Pilei applanate, dimidiate or semicircular; basidiospores >3.7 μm wide.........................................................................................................47

47. Pileal surface encrusted.................................................................48

47. Pileal surface not encrusted..........................................................49

48. Basidiospores 3.9–4.5 μm long; Central American species......*F. centroamericanus* Y.C. Dai et al.

48. Basidiospores 4.6–5.1 μm long; Asian species.......*F. imbricatus* L.W. Zhou

49. Pores 9–11 per mm..................*F. costaricense* Y.C. Dai & Vlasák

49. Pores 7–9 per mm.....................................*F. nilgheriensis* (Mont.) Bondartseva & S. Herrera

**Key to species of *Pyrrhoderma***

1. Hyphoid setae absent.........................................................................2

1. Hyphoid setae present........................................................................3

2. Pores 5–6 per mm; basidiospores 6–7 μm long...........................*P. adamantinum* (Berk.) Imazeki

2. Pores 3–5 per mm; basidiospores 4–4.5 μm long........................*P. thailandicum* L.W. Zhou & Y.C. Dai

3. Pores 2–4 per mm; dissepiments lacerate...............*P. luteofulvum* (Cleland & Rodway) Y.C. Dai & F. Wu

3. Pores 6–9 per mm; dissepiments entire..........................................4

4. Hymenial setae present..............................................*P. yunnanense* L.W. Zhou & Y.C. Dai

4. Hymenial setae absent........................................................................5

5. Basidiocarps annual.....................................................*P. hainanense* L.W. Zhou & Y.C. Dai

5. Basidiocarps perennial.......................................................................6

6. Basidiospores 3–3.6 µm wide................................................*P. nigra*

6. Basidiospores 2–2.4 μm wide............................................................7

7. Contextual hyphae interwoven, basidiospores oblong-ellipsoid, 3.2–4.3 μm long...............*P. lamaoense* (Murrill) L.W. Zhou & Y.C. Dai

7. Contextual hyphae regularly arranged, basidiospores ellipsoid, 2.6–3.3 μm long.....................*P. sublamaensis* (Lloyd) Y.C. Dai & F. Wu

## Data availability statement

The datasets presented in this study can be found in online repositories. The names of the repository/repositories and accession number(s) can be found in the article/supplementary material.

## Author contributions

MZ, YY and JV coordinated the project and designed the experimental plan. MZ and YY analyzed the data with help from XHJ and JV. MZ, YY, HGL, KM and JV collected the samples from the field. MZ, X-HJ and YY writing the original draft preparation. MZ, X-HJ, YY and JV review and editing the manuscript. YY and JV acquire funding. All authors contributed to the article and approved the submitted version.
